# CAREGIVER CHALLENGES: SOCIAL SUPPORT FOR DEMENTIA CARE OF OLDER ADULTS DURING HOSPICE ENROLLMENT

**DOI:** 10.1093/geroni/igad104.3210

**Published:** 2023-12-21

**Authors:** Hannah Cho, Justine Sefcik, Karla Washington, Debra Parker Oliver, George Demiris

**Affiliations:** University of Pennsylvania, Philadelphia, Pennsylvania, United States; Drexel University College of Nursing and Health Professions, Philadelphia, Pennsylvania, United States; Washington University in St. Louis, St. Louis, Missouri, United States; Washington University in St. Louis, St. Louis, Missouri, United States; University of Pennsylvania, Philadelphia, Pennsylvania, United States

## Abstract

Every year, many ADRD patients rely on informal caregivers for providing care during hospice enrollment. Driven by dedication, these caregivers play a vital role. However, their responsibilities intensify over time, leading to emotional strain. Inadequate support causes burnout and stress. Limited research targets the unique needs of caregivers of ADRD patients enrolled in hospice. The purpose of this study was to describe the caregivers’ experiences regarding social support while providing care for older adults with ADRD in hospice. A qualitative descriptive approach was taken with semi-structured interviews of 20 caregivers of ADRD hospice patients (85% female; mean age [SD]: 63.75 [12.1]), including both Caucasian (70%) and African American (30%) individuals. Through content analysis, we identified three themes that described caregivers’ experiences with social support: 1) Changes in social life (e.g., homebound, stress, and family conflicts), 2) Opportunities for social connection (e.g., social support systems with family, friends), 3) Communication Modalities. These findings offer crucial insights into the diverse range of experiences caregivers face regarding social support. Additionally, it highlights the significance of caregivers needing assistance to sustain connections, take essential breaks, and adapt to shifts in their social environments. The challenges they confront and the positive elements of the support they receive are brought to light through this study. Further research into effective communication modalities and respite services could significantly enhance caregivers’ support.

